# Angiogenesis: From Chronic Liver Inflammation to Hepatocellular Carcinoma

**DOI:** 10.1155/2010/272170

**Published:** 2010-05-27

**Authors:** Paloma Sanz-Cameno, María Trapero-Marugán, María Chaparro, Evan Anthony Jones, Ricardo Moreno-Otero

**Affiliations:** ^1^Laboratory of Investigation, Molecular Biology Unit, Hospital Universitario de la Princesa, Universidad Autónoma de Madrid, 28006-Madrid, Spain; ^2^Hepatology Unit, Hospital Universitario de la Princesa, Universidad Autónoma de Madrid, 28006 Madrid, Spain; ^3^CIBERehd, Instituto de Salud Carlos III, 28006 Madrid, Spain

## Abstract

Recently, new information relating to the potential relevance of chronic hepatic inflammation to the development and progression of hepatocellular carcinoma (HCC) has been generated. Persistent hepatocellular injury alters the homeostatic balance within the liver; deregulation of the expression of factors involved in wound healing may lead to the evolution of dysplastic lesions into transformed nodules. Progression of such nodules depends directly on the development and organization of a vascular network, which provides the nutritional and oxygen requirements to an expanding nodular mass. Angiogenic stimulation promotes intense structural and functional changes in liver architecture and physiology, in particular, it facilitates transformation of dysplasia to nodular lesions with carcinogenic potential. HCC depends on the growth and spreading of vessels throughout the tumor. Because these vascular phenomena correlate with disease progression and prognosis, therapeutic strategies are being developed that focus on precluding vascular expansion in these tumors. Accordingly, an in-depth study of factors that promote and support pathological angiogenesis in chronic hepatic diseases may provide insights into methods of preventing the development of HCC and/or stimulating the regression of established HCC.

## 1. Introduction

In recent years an appreciable worldwide increase in the incidence of tumors, such as hepatocellular carcinoma (HCC), has occurred in association with changes in socioeconomic development, which include potentially relevant changes in lifestyles [1]. Development of HCC is known to be triggered by factors that lead to chronic hepatic injury and deregulation of the normal process of wound healing, which promote persistent stimulation of profibrotic and proangiogenic processes that lead to significant structural changes in the liver and functional changes in hepatic physiology [2].

 Formation of new blood vessels (angiogenesis) is closely related to the development of HCC. Indeed, HCC is a highly vascularized tumor, a feature that has implications for investigative procedures applied for its detection [3]. Among features of the vasculature of the liver, not found in other tissues, are the hepatic sinusoids, characteristics of which include the presence of hepatic sinusoidal endothelial cells (LSECs) that possess distinctive fenestrations and pericytes or hepatic stellate cells (HSCs); sinusoids have the ability to synthesize liver-specific factors, such as angiopoietin-like 3 (Angpl3). Angiogenesis in HCC depends on the same fundamental principles of activation, proliferation and migration of endothelial cells that occur in other tumors and diseases in which enhanced angiogenesis occurs [4]. 

**Figure 1:**
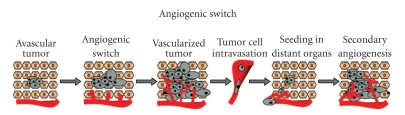
Angiogenic switch: transformed cells proliferate as an avascular nodule until they reach a certain size. Angiogenic switch enables exponential tumor growth and facilitates dissemination of tumor cells to secondary locations, where pathological angiogenesis may again be initiated.

 Neoplastic tissue requires a supply of oxygen and nutrients. Thus, avascular solid tumors only grow to a certain size and then undergo regression, if their metabolic demands are not met [5]. For continued growth, it is necessary for a tumor to orchestrate the formation of a functioning system of blood vessels, which allows the delivery of metabolites (including growth factors) and cells (immunological cells and other cellular precursors) to the tumor environment. This process involves neo-vascularization of the tumor or tumor angiogenic switch [6, 7]. Once the requirements for tumor growth are met as a result of angiogenesis, the tumor mass then becomes restricted by surrounding normal tissue, which degenerates and is replaced by tumor tissue. These phenomena stimulate further immune-driven angiogenesis; formation of more blood vessels tends to ameliorate tissue damage. Moreover, spatiotemporal deregulation of pathological angiogenesis may lead to newly formed vessels having abnormal architecture and function; the frequently immature and anomalous characteristics of tumor vasculature tend to facilitate the spread of tumor cells [8]. If disseminated tumor cells become located in other tissues, the whole process of tumorigenesis may reoccur with the result that secondary tumors are generated (Figure 1).

## 2. Factors That Stimulate Angiogenesis

Hypoxia and inflammation are the main factors that stimulate angiogenesis. Hypoxia promotes angiogenesis as a consequence of signalling mediated by hypoxia-inducible transcription factors [9]. Inflammation increases vascular permeability and promotes chemokine-mediated recruitment of monocytes, macrophages, platelets, mast cells and other leukocytes that can synthesize angiogenic cytokines and growth factors [10, 11].

**Figure 2:**
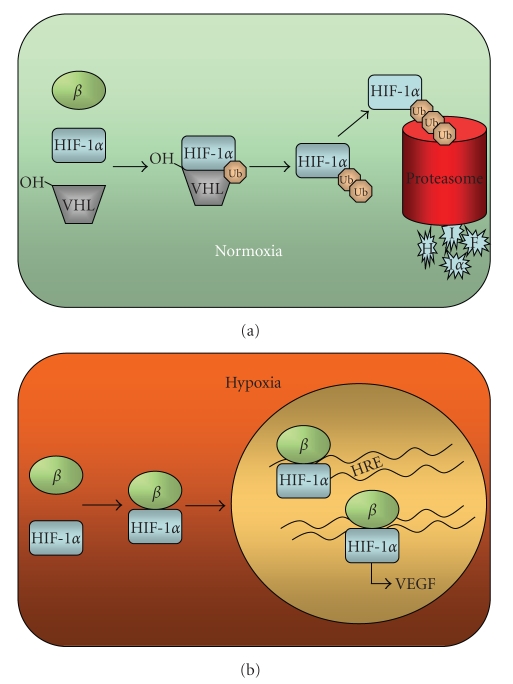
Oxygen-dependent regulation of HIF-1*α*: in normal oxidative conditions, HIF-1*α* is hydroxylated and it becomes ubiquitinated by VHL. Subsequently, HIF-1*α*  is degraded by the proteasome. In contrast, low oxygen tension leads to stabilization of HIF-1 and its interaction with beta subunit, which triggers translocation to the nucleus and modulation of the transcription of diverse genes that are involved in the response to hypoxia.

 Specifically, hypoxia impairs the hydroxylation of HIF-1alpha and hence, reduces its subsequent degradation by proteasome. Consequently, HIF-1alpha accumulates in the cytosol, thereby facilitating its interaction with beta subunits and translocation to the nucleus, with the consequent modulation of the expression of many angiogenesis-promoting genes that contain hypoxia-responsive elements (HREs) (Figure 2).

**Figure 3:**
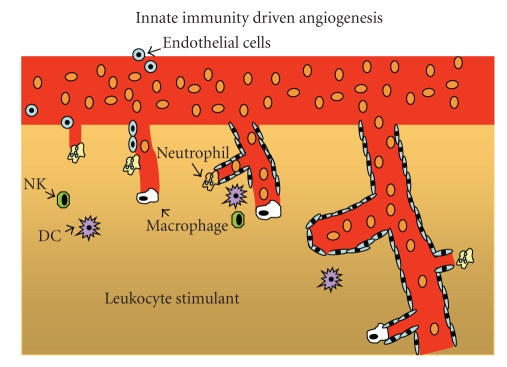
Innate immunity-driven angiogenesis: immune cells, mostly neutrophils and monocyte-macrophages, mediate initial tunnel formation in certain models of angiogenesis. Other myeloid cell types, such as dendritic cells (DCs) and natural killer cells (NK), produce angiogenic factors that attract endothelial cells that become essential components of developing blood vessels. Adapted from Noonan et al., 2008.

 Furthermore, tissue injury triggers an immune response, in which immune cells in peripheral blood extravasate into the damaged tissue, where they mediate restoration of tissue homeostasis. However, if the injury and associated inflammation persist, continuing endothelial activation would promote the infiltration of immune factors and cells into the focus of inflammation; this process may stimulate the proliferation and migration of endothelial cells, the prerequisites for the generation of new blood vessels (Figure 3) [12]. Thus, a close relationship between chronic inflammation, angiogenesis and cancer, recently the subject of several reviews [13–17], would be expected.

## 3. Phases of Angiogenesis

The formation of new functional blood vessels depends on precise regulation of the molecular effectors that promote the different processes involved [13, 14].

Endothelial cell budding is facilitated by vasodilatation, loosening of interendothelial contacts and leakage from pre-existing vessels. These phenomena allow extravasation of plasma proteins that, together with ECM components, facilitate the laying down of a provisional scaffold for migrating endothelial cells (EC). Nitric oxide (NO), the angiogenic properties of which have been well characterized [18–20], is the main factor responsible for vasodilatation. Vascular endothelial growth factor (VEGF) increases vascular permeability. The basement membrane (mainly collagen type IV and laminin) and the ECM (collagen type I and elastin) must undergo degradation to allow subsequent migration and proliferation of EC. This process is mediated by specific proteinases, which include plasminogen activator. Proteolysis of ECM leads to the exposure of cryptic epitopes and release of ECM-embedded factors that promote migration and proliferation of EC [21, 22]. 

ECs proliferate in response to growth factors secreted by themselves or by surrounding cells, such as hepatic stellate cells, leukocytes, hepatocytes and Kupffer cells. The most thoroughly characterized of these growth factors is VEGF, which is a multifunctional protein that binds to two tyrosine kinase receptors that are designated kinase insert domain receptor (KDR) and fms-like tyrosine kinase receptor (Flt-1) [23]. VEGF, whose promoter contains hypoxia-inducible factor-responsive elements (HREs), plays a crucial role virtually in all pathological situations in which angiogenesis occurs. The therapeutic potential of strategies aimed at blocking its mechanism of action is currently being evaluated [24].

A VEGF-induced increase in vascular permeability facilitates interchange of metabolites and cells between peripheral blood and surrounding tissues. The vasculature constitutes a structural and functional barrier to the transfer of cells and solutes, which may traverse vascular endothelium by paracellular and/or transcellular routes [25]. The former route implies the rupture of intercellular junctions, while the latter route involves the organization of caveolae or vacuolar vesicles to generate an intracellular conduit.

 VEGF can increase the permeability of the endothelium by enhancing both routes of transfer across vascular endothelium. Signalling mediated by VEGFR2 promotes the phosphorylation of vascular endothelial cadherin (VE-Cadh), which impairs its binding to the actin cytoskeleton and, hence, leads to deterioration of intercellular junctions. In addition, this phenomenon is enhanced by VEGF-mediated dissociation of the VE-PTP phosphatase of VE-Cadh. Furthermore, the increase of VE-Cadh endocytosis, as well as the improvement in pathways of transcytosis mediated by VEGF, may account for associated vascular integrity and modulation of permeability [26]. As the delivery of antiangiogenic and antitumor drugs to subendothelial tumor tissue is promoted by increased vascular permeability, methods of modulating the transcellular transfer of drugs are being studied in depth with the aim of enhancing the efficacy of pharmacotherapies for cancer.

**Figure 4:**
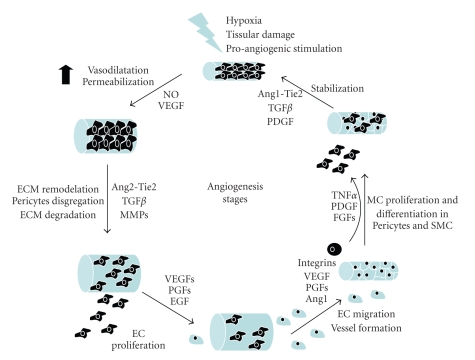
Stages of angiogenesis: angiogenic stimuli, by promoting production of NO and VEGF, lead to dilatation and increased permeability of blood vessels. Subsequently, other factors, particularly Ang-1, TGF-*β*, and MMPs, facilitate degradation of basement membrane and extracellular matrix and detachment of pericytes. Consequently, angiogenic stimuli facilitate proliferation and migration of endothelial cells. In addition, mesenchymal cells proliferate and differentiate into pericytes and smooth muscle cells that contribute to the stabilization of the new blood vessels.

In addition to VEGF, proliferation of EC may be stimulated by other growth factors, such as acidic and basic fibroblast growth factors (aFGF, and bFGF), hepatocyte growth factor (HGF) and transforming growth factor (TGF) [27–29]. An orderly proliferation of EC leads to the formation of a lumen [30, 31]. A structured 3-dimensional network of vessels of uniform size then develops, with its organization being regulated by mechanisms that involve signalling pathways for determination of branching and formation of basement membrane and ECM components, as well as migration of cells and their differentiation. Maturation of nascent vessels requires recruitment of pericytes, and formation of a new basement membrane and ECM to provide structural stabilization [31]. Both physical forces and specific different molecules contribute to these processes (Figure 4).

Additionally to the induction of proliferation of EC, effective angiogenesis also requires stabilization of nascent blood vessels, and formation of interendothelial cell junctions and lumens. Angiopoietin-1 (Ang-1) stabilizes nascent vessels by binding to the Tie-2 receptor, thereby modulating junctional molecules [32] and facilitating communication between EC and mural cells [33]. However, an excess of Ang-1 increases stiffness and inhibits branching of vessels. Angiopoietin-2 (Ang-2) may mediate opposing effects: in the absence of VEGF, it antagonizes Ang-1, thereby destabilizing vessels, inducing death of EC and leading to regression of vessels; in the presence of VEGF, however, it facilitates the branching of vessels [33, 34].

 Zhang et al. [35] evaluated the expression of angiogenic factors in specimens of HCC and nonneoplastic hepatic tissue. They found a higher expression of Ang-2, and lower expression Ang-1 in HCC than in nonneoplastic tissue, suggesting that these molecules may play a role in angiogenesis associated with carcinogenesis and progression of HCC. Pappeti and Herman have reported that pathological angiogenesis associated with tumors is different from that associated with physiological processes [36]. Under normal conditions vascular inactivity and stabilization are mediated by Ang-1, Ang-2, and Tie-2; in pathological angiogenesis an abnormal Ang-2/Ang-1 ratio, in the presence of VEGF, plays a critical role in the transformation of noncancerous liver tissue to HCC by initiating early neovascularization. Vajkoczy et al. [37] reported that the earliest stage of tumor development is initiated by VEGF, VEGF receptor-2 and Ang-2 interacting with host vessels. Therefore, augmented expression of Ang-2, and downregulation of Ang-1, acting via the Tie2 receptor in the presence of VEGF, play important roles in initiating early neovascularization and transformation of noncancerous hepatic tissue to HCC.

## 4. Physiological Angiogenesis

Experimental animal models of liver regeneration after partial hepatectomy have enabled the development of functional sinusoids to be studied. Resection of two thirds of the total liver mass results in stimulation of quiescent hepatocytes, and activation of numerous transcription factors that modulate many genes involved in the proliferation of hepatocytes [38–40]. An exponential increase in the number of hepatocytes leads to definitive architectural and functional changes in the liver; avascular clusters of cells are generated that induce the differentiation and subsequent replication of the other types of hepatic cells, notably liver sinusoidal endothelial cells (LSECs), hepatic stellate cells (HSCs), and other EC precursors mobilized from the bone marrow. All of these different types of cell migrate and interact with the emerging mass of hepatocytes; they augment the metabolic functions, including energy metabolism, of hepatocytes and provide structural support of the maturing hepatic parenchyma. Subsequently, fullyfunctional sinusoids are generated about six to eight days after hepatectomy [41, 42].

## 5. Pathological Hepatic Angiogenesis

**Figure 5:**
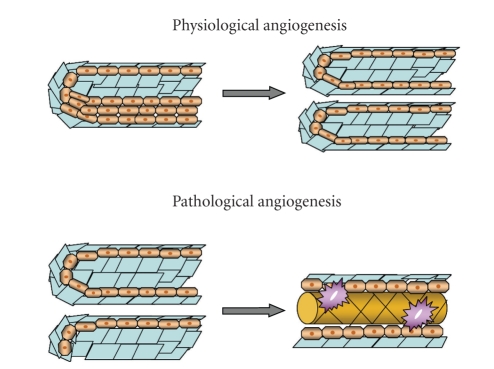
Hepatic angiogenesis: during liver regeneration, newly formed sinusoids have normal architecture consistent with their physiological roles. In contrast, pathological hepatic angiogenesis leads to the capillarization of hepatic sinusoids with the result that the structure of sinusoids becomes distorted and characteristic endothelial fenestrations are lost.

While physiological hepatic angiogenesis during liver regeneration leads to the formation of new functional sinusoids, pathological angiogenesis, which occurs in many chronic liver diseases, is characterized by the appearance of capillarized vascular structures [16, 43] (Figure 5). Such anomalous intrahepatic vascular organization impairs the physiological interplay between sinusoids and parenchymal cells. Hepatic injury is exacerbated by enhanced activation of immunological responses and generation of more intense hypoxic areas. 

Most chronic liver diseases are characterized by diffuse, chronic processes of inflammation, necrosis and fibrosis [2]. Altered mechanisms of chronic wound healing in response to persistent liver injury lead to an imbalance in the expression of many cytokines, growth factors and metalloproteinases (MMPs), which deregulate pathways of physiological angiogenesis and, hence, lead to structural and functional hepatic disorganization [44]. These phenomena induce accumulation of an excess of extracellular matrix, the composition of which becomes altered by the deposition of fibrillar collagen (type I) rather than physiological sinusoidal collagen (type IV). Subsequently, the characteristic fenestrations of LSEC become blocked, and the physiological interchange of oxygen and metabolic factors is hampered. The deposition of fibrous tissue increases resistance to blood flow and, consequently, impairs the delivery of oxygen to the parenchyma; the resulting tissue hypoxia exacerbates tissue injury.

 Our group described the existence of abnormal vascular architecture in the liver of patients with autoimmune hepatitis and primary biliary cirrhosis; these intrahepatic structural changes were associated with an increase in the expression of VEGF and angiopoietins [45]. Similar changes in VEGF and angiopoietins have been reported in the serum and liver tissue of patients with other chronic liver diseases, including chronic hepatitis B and, more recently, chronic hepatitis C [34, 46]. 

The development of HCC has long been related to chronic hepatocellular injury. Malignant transformation of hepatocytes may take place in regenerative nodules that undergo dysplastic changes. However, a potential contribution of hepatocytic precursors cannot be dismissed. Uncontrolled proliferation of hepatocytes generates hypoxic hepatic nodules that promote the stimulation of mechanisms that mediate the angiogenic switch, a phenomenon that has both diagnostic and therapeutic relevance [47–49]. 

 In addition to the development of pharmacologic interventions directed at modulating many of the signalling routes involved in cell proliferation, survival and invasion [50], the hypervascular characteristics of HCC have also encouraged therapeutic approaches that focus on inhibiting progression of the vascular network present in incipient malignant nodules and the distinctive tumor vasculature in established HCC [51, 52]. In many advanced malignancies, effective delivery of therapeutic drugs to the tumor is limited by specific complex features of tumor tissue. Recently, delivery of pharmacologic agents to malignant nodules has been enhanced by adopting measures that facilitate the transcellular route of molecular trafficking [53].

 In summary, the relevance of chronic hepatocellular injury to the development of HCC has been emphasized. In particular, the significance of angiogenesis in the progression of chronic hepatic disease to hepatic malignancies has been highlighted. A role for deregulation of different stages of angiogenesis in the establishment of advanced tumours and their subsequent dissemination to other tissues has become recognized. Accordingly, the in-depth study of regulatory mechanisms that mediate angiogenesis in pathological conditions would appear to be indicated. One specific aim of such an initiative would be to augment the efficacy of currently available therapies for chronic (inflammatory) hepatic disease and HCC.
